# DNA Methylation Profile in Human Cord Blood Mononuclear Leukocytes From Term Neonates: Effects of Histological Chorioamnionitis

**DOI:** 10.3389/fped.2020.00437

**Published:** 2020-08-04

**Authors:** Gina Fong, Suhita Gayen nee' Betal, Swati Murthy, Michael Favara, Joanna S. Y. Chan, Sankar Addya, Thomas H. Shaffer, Jay Greenspan, Vineet Bhandari, Dongmei Li, Irfan Rahman, Zubair H. Aghai

**Affiliations:** ^1^Neonatology, Thomas Jefferson University/Nemours, Philadelphia, PA, United States; ^2^Department of Pathology, Thomas Jefferson University, Philadelphia, PA, United States; ^3^Laboratory of Cancer Genomics, Thomas Jefferson University, Philadelphia, PA, United States; ^4^Section of Neonatology, Department of Pediatrics, Cooper University Hospital, Camden, NJ, United States; ^5^Department of Environmental Medicine, University of Rochester Medical Center, Rochester, NY, United States

**Keywords:** epigenetics, leukocytes, histological chorioamnionitis, differential DNA methylation, immune modulation

## Abstract

**Background:** Histological chorioamnionitis (HCA) is an infection/inflammation of fetal membranes and complicates 5.2–28.5% of all live births. Exposure to HCA can have long-term consequences including abnormal neurodevelopment and an increased risk for allergic disorders and asthma later in childhood. HCA may incite epigenetic changes, which have the potential to modulate both the immune and neurological systems as well as increase the risk of related disorders later in life. However, there is limited data on the impact of HCA on epigenetics, in particular DNA methylation, and changes to immune and neurological systems in full-term human neonates.

**Objective:** To determine differential DNA methylation in cord blood mononuclear leukocytes from neonates exposed to HCA.

**Methods:** Cord blood was collected from 10 term neonates (5 with HCA and 5 controls without HCA) and mononuclear leukocytes were isolated. Genome-wide DNA methylation screening was performed on Genomic DNA extracted from mononuclear leukocytes.

**Results:** Mononuclear leukocytes from cord blood of HCA-exposed neonates showed differential DNA methylation of 68 probe sets compared to the control group (44 hypermethylated, 24 hypomethylated) with a *p* ≤ 0.0001. Several genes involved in immune modulation and nervous system development were found to be differentially methylated. Important canonical pathways as revealed by Ingenuity Pathway Analysis (IPA) were CREB Signaling in Neurons, FcγRIIB Signaling in B Lymphocytes, Cell Cycle: G1/S Checkpoint Regulation, Interleukin-1, 2, 3, 6, 8, 10, 17, and 17A signaling, p53 signaling, dopamine degradation, and serotonin degradation. The diseases and disorders picked up by IPA were nervous system development and function, neurological disease, respiratory disease, immune cell trafficking, inflammatory response, and immunological disease.

**Conclusions:** HCA induces differential DNA methylation in cord blood mononuclear leukocytes. The differentially methylated genes may contribute to inflammatory, immunological and neurodevelopmental disorders in neonates exposed to HCA.

## Background

Chorioamnionitis (CA) is an infection/inflammation of the placenta and fetal membranes; it complicates up to 5.2% of live births (1) with histological CA (HCA) being found in 23.6% to 28.7% of term births with spontaneous labor (2, 3). HCA has been linked to several common chronic diseases of childhood: cerebral palsy, developmental delay, asthma and allergic disorders (4–13). The exact mechanism of these associations is unclear. Exposure to perinatal inflammation may stimulate epigenetic changes that alter immune function and subsequently contribute to these pathologies.

Prior studies have shown an association between perinatal environment, including epigenetic changes, and disease later in life (14, 15). Epigenetic changes can occur through multiple mechanisms including histone modification, DNA methylation, and RNA interference. DNA methylation is an enzyme-mediated change to DNA in which a methyl group is added to cytosine at CpG sites, an important mechanism of transcriptional regulation (16). Increased methylation results in reduced transcription of adjacent genes while decreased methylation results in increased transcription of adjacent genes. HCA is known to be associated with epigenetic changes via DNA methylation of key developmental genes in preterm neonates (17). Additionally, DNA methylation has been shown to be altered in placentas and fetal membranes with HCA (18).

The effects of HCA on DNA methylation in full term neonates are not yet known and may be an important mechanism in increasing susceptibility to disease later in life. Our objective was to evaluate the DNA methylation patterns of cord blood mononuclear leukocytes from neonates exposed to HCA compared with healthy controls.

## Materials and Methods

### Ethical Approval: Human Study Protocol and Institutional Biosafety Approvals

All human protocols and procedures described in this study were approved by the Institutional Review Board of Thomas Jefferson University Hospital. All experiments performed in this study were approved by the Nemours Institutional Biosafety Committee. The Institutional Review Board waived informed consent as the study was performed on discarded blood and placental tissue samples.

### Study Design

This is a prospective observational study to examine differential DNA methylation in mononuclear leukocytes isolated from cord blood of term neonates born to mothers with HCA compared to those without HCA. Samples of cord blood and fetal membranes were collected from term neonates (37–40 weeks of gestation). Exclusion criteria included maternal infections other than HCA, gestational diabetes, hypertension, major congenital/chromosomal anomalies, and intrauterine growth restriction.

### Cord Blood Collection and Isolation of Leukocytes

The umbilical cord was wiped with 70% alcohol immediately after delivery and cut at the placental side of the clamp. Cord blood was collected in sterile EDTA tubes, mixed thoroughly, and evaluated for blood clots. Mononuclear leukocytes were isolated by Ficoll-Paque Plus density gradient (GE Healthcare Biosciences, Pittsburgh, PA), following the manufacturer's protocol and the method described previously by our group (19). Two million packed cells per vial were saved at −80°C for DNA isolation and global DNA methylation analysis.

### Fetal Membrane Collection, Processing, Staining, and Diagnosis of HCA

Fetal membrane tissues were washed with cold PBS and fixed in 10% neutral buffered formalin for 24–48 h. Membrane pieces were then processed and paraffin embedded in Histoplast LP (Thermo Fisher Scientific, Fremont, CA) following the detailed method of Gayen nee' Betal et al. (19). Tissue samples were processed using standard operating procedures at the Nemours Histochemistry and Tissue Processing Core (Nemours, Wilmington, DE). The tissue samples were then examined by a blinded pathologist and classified either HCA (placental membranes score ≥ stage 1) or no HCA (no histological inflammatory changes in fetal membranes) (20) (Supplemental Figure 1).

### DNA Isolation and Global DNA Methylation Analysis

Genomic DNA was isolated using QIAamp DNA Mini kit (Qiagen, Germantown, MD). DNA was quantified on a Nanodrop ND-2000 spectrophotometer (Thermo Fisher Scientific, Waltham, MA), and the DNA quality was assessed by an Agilent 2200 TapeStation (Agilent Technologies, Palo Alto, CA). Analysis of genome-wide DNA methylation was performed using the Infinium HumanMethylationEPIC BeadChip array (Illumina, San Diego, CA) at the Children's Hospital of Philadelphia following the standard protocol provided by Illumina. In brief, 500 ng of genomic DNA was bisulfite-converted using the EZ DNA Methylation Kit (Zymo Research, Orange, CA) according to the manufacturer's protocol. The Illumina iScan Reader was used to analyze the image and data from EPIC BeadChip. Data processing was performed with Illumina GenomeStudio software.

### Quality Control and Statistical Approaches for DNA Methylation Analysis

Quality control (QC) was performed on DNA methylation data to filter out probes with missing methylation values in any samples and probes with at least 75% of samples having detected a *p* > 0.05. 866,836 probes remained for further analysis after QC filtering. DNA methylation β-values were normalized using the SWAN normalization method in minfi package in R/Bioconductor (21, 22). After normalization, DNA methylation sites with maximum methylation level below 0.15 were further filtered out to remove probes with low DNA methylation levels from next step differential analysis. A general linear model approach was then used to detect the difference in methylation levels between healthy neonates and neonates born to mothers with HCA using the limma package in R/Bioconductor (23). The moderated t-statistics with empirical Bayes approach was used to estimate the differences in methylation levels between healthy neonates and those born to mothers with HCA. Top DNA methylation sites were selected by test statistics and *p*-values (with raw *p* < 0.0001) from the two group comparisons. A Manhattan plot was used to show the selected top DNA methylation sites and their chromosome positions. A volcano plot of all DNA methylation sites was generated to show both their *p*-values and fold changes from the two group comparisons. A heatmap of methylation levels from top DNA methylation sites was generated to show the DNA methylation pattern differences between groups. All plots were generated using the program “R.”

## Results

Genome-wide DNA methylation was performed on mononuclear leukocytes from cord blood of 10 term neonates. Five neonates had evidence of HCA by placental histopathology (referred as “HCA Group”) and five neonates had no evidence of HCA (referred as “control group”).

### Differential DNA Methylation

**Figure 1:**
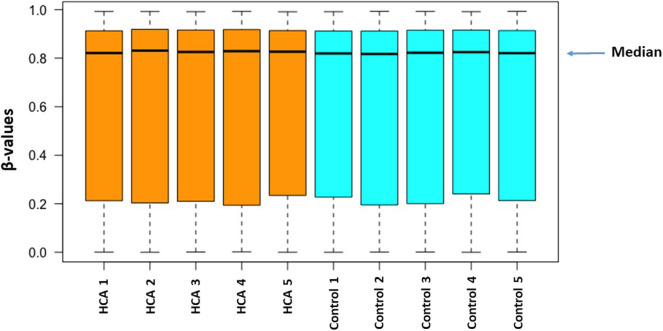
The distribution of methylation level (β-values) among all of the samples from the two groups.

DNA methylation levels are represented by β-values. The β-value is the ratio of the methylated probe signal intensity to the total locus intensity. The β-values range from 0 to 1 where 0 indicates unmethylated and 1 indicates fully methylated. A boxplot (Figure 1) was generated from β-values of all probe sets from the 10 samples to describe distribution of the pre-processed DNA methylation level. The interquartile range of the β-values was 0.2–0.9. Although the spread of β-values varied slightly within each group, no significant differences between groups were observed.

Comparison of the HCA group genome-wide DNA methylation data with the control group using Illumina GenomeStudio software identified 68 differentially methylated probe-IDs with a *p* ≤ 0.0001. Of these, 44 probe-IDs were significantly hypermethylated and 24 probe-IDs were significantly hypomethylated (Table 1). The top 10 hyper and hypomethylated probe-IDs along with their corresponding gene names based on *p*-values are listed in Tables 2, 3. Notable hypermethylated probe-IDs with gene names included AGR2 (Anterior Gradient Protein 2), which plays a role in asthma and mucin production (24), and OTX2 (Orthodenticle Homeobox 2), which interacts with nitric oxide (25). The top hypomethylated probe-IDs with gene names included MIER2 (Mesoderm Induction Early Response Protein 2), which regulates histone deacetylases (26), and PPP4C (Protein Phosphatase 4 Catalytic Subunit), a regulator of T-regulatory cells (27).

**Table 1:** Differentially methylated CpGs with exposure to histological chorioamnionitis (*p* ≤ 0.0001).

**CpG site**	**Gene symbol**	**Methylation status**	**Chromosome**	**Strand**	**Test statistics**	**Raw *p*-value**	**Island name**	**Relation to island**	**Reference gene group**
cg19448065	VGLL4; VGLL4	Hypermethylated genes	chr3	+	15.189	8.54E-08		OpenSea	Body; Body
cg00995577	AGR2		chr7	+	10.897	1.53E-06		OpenSea	TSS1500
cg12363975	AGAP1; AGAP1; AGAP1		chr2	+	10.709	1.78E-06		OpenSea	Body; Body; Body
cg26386257			chr7	+	10.633	1.89E-06		OpenSea	
cg09304034	SMIM15;CTC-436P18.1		chr5	–	9.534	4.75E-06	chr5:60457840-60458654	N_Shore	5′UTR;TSS1500
cg08049519			chr15	–	9.363	5.53E-06	chr15:81410715-81411067	S_Shore	
cg16380389			chr16	–	9.043	7.39E-06	chr16:90069762-90070883	Island	
cg04381379	CELF4; CELF4; CELF4;CELF4		chr18	+	8.385	1.38E-05		OpenSea	Body; Body; Body; Body
cg27344140			chr3	+	8.329	1.45E-05		OpenSea	
cg08258922			chr14	–	8.129	1.77E-05	chr14:103745232-103746271	Island	
cg02086467	OTX2		chr14	–	7.855	2.34E-05	chr14:57274607-57276840	Island	5′UTR
cg18196614	MCM8-AS1		chr20	+	7.710	2.72E-05	chr20:5986399-5987297	N_Shelf	Body
cg04040779	NUP160		chr11	–	7.439	3.62E-05		OpenSea	Body
cg14508517	NAALADL2		chr3	–	7.340	4.03E-05		OpenSea	Body
cg15057251	MAPRE2; MAPRE2; MAPRE2; MAPRE2		chr18	–	7.306	4.18E-05	chr18:32621215-32621689	Island	TSS200; 5′UTR; TSS200; Body
cg06977182	SEMA6A; SEMA6A		chr5	–	7.296	4.22E-05		OpenSea	Body; Body
cg04423025	PRCP; PRCP		chr11	+	7.292	4.24E-05		OpenSea	Body; Body
cg18426912	NDUFAF5; NDUFAF5; NDUFAF5		chr20	–	7.190	4.74E-05	chr20:13765166-13765909	S_Shelf	Body; Body; Body
cg07417772	GOLIM4; GOLIM4		chr3	+	7.155	4.93E-05		OpenSea	Body; Body
cg21147013	LOC102467224		chr5	+	7.126	5.09E-05		OpenSea	Body
cg10176510	NDUFAF4		chr6	–	7.124	5.10E-05		OpenSea	3′UTR
cg18159802			chr11	+	7.117	5.14E-05		OpenSea	
cg07891737	MIR519D; MIR517A		chr19	+	7.113	5.16E-05		OpenSea	TSS1500; Body
cg16510851			chr3	+	7.109	5.18E-05		OpenSea	
cg05574878	MIR1912; HTR2C		chrX	–	6.959	6.13E-05		OpenSea	TSS1500; 5′UTR
cg07277182	FANCM; FANCM		chr14	+	6.958	6.13E-05		OpenSea	Body; Body
cg03508395			chr14	+	6.947	6.21E-05	chr14:104927441-104928653	S_Shore	
cg00080312	SACM1L		chr3	–	6.932	6.32E-05	chr3:45730615-45731294	N_Shore	TSS1500
cg16077353	STXBP5-AS1		chr6	–	6.904	6.52E-05		OpenSea	Body
cg26548811	LOC101929239; PACRG; PACRG; PACRG		chr6	–	6.903	6.52E-05		OpenSea	Body; Body; Body; Body
cg12540696	FMN2; FMN2		chr1	–	6.889	6.63E-05		OpenSea	Body; Body
cg14541152	SCAMP5		chr15	–	6.876	6.73E-05	chr15:75315855-75316178	N_Shelf	3′UTR
cg22184818	TTC29; TTC29		chr4	–	6.873	6.75E-05		OpenSea	Body; Body
cg06028808			chr11	+	6.840	7.01E-05		OpenSea	
cg00558645			chr1	+	6.814	7.22E-05		OpenSea	
cg17130789	SLC11A1		chr2	–	6.806	7.29E-05	chr2:219255949-219256210	Island	Body
cg07480541	LRCH2; LRCH2		chrX	+	6.706	8.17E-05		OpenSea	Body; Body
cg02021337	KLHL24		chr3	+	6.686	8.36E-05		OpenSea	3′UTR
cg05063703			chr9	+	6.673	8.49E-05		OpenSea	
cg18189005	CACNA1A; CACNA1A; CACNA1A; CACNA1A; CACNA1A		chr19	+	6.669	8.53E-05		OpenSea	Body; Body; Body; Body; Body
cg22396161	ATP7A		chrX	–	6.667	8.55E-05		OpenSea	3′UTR
cg17341671			chr1	+	6.555	9.73E-05		OpenSea	
cg03514545	SYT6		chr1	+	6.551	9.79E-05	chr1:114696886-114697185	N_Shore	TSS1500
cg26013396	SGPP1		chr14	–	6.547	9.83E-05	chr14:64194241-64195049	N_Shelf	Body
cg02417264	RAMP3	Hypomethylated genes	chr7	+	−6.570	9.56E-05	chr7:45197181-45197807	Island	Body
cg12693181			chr11	–	−6.613	9.10E-05	chr11:18067716-18067928	N_Shelf	
cg19315263	ZNF471		chr19	+	−6.615	9.08E-05	chr19:57018743-57019506	S_Shore	5′UTR
cg12313868			chr11	–	−6.718	8.06E-05		OpenSea	
cg15343100	CCDC68; CCDC68		chr18	–	−6.735	7.90E-05	chr18:52626517-52626849	N_Shore	TSS1500; 5′UTR
cg16408982			chr14	+	−6.805	7.30E-05	chr14:103522988-103524431	S_Shelf	
cg26933865	PPP4C		chr16	+	−6.904	6.52E-05	chr16:30087236-30087842	N_Shore	TSS1500
cg12743398	SULT1A2		chr16	+	−6.953	6.17E-05	chr16:28611946-28612388	N_Shelf	TSS1500
cg18901644	GPR78		chr4	–	−7.030	5.66E-05	chr4:8582036-8583364	Island	TSS200
cg01293188			chr4	+	−7.117	5.14E-05		OpenSea	
cg19870898			chr14	–	−7.124	5.10E-05	chr14:103673773-103674343	S_Shelf	
cg24376295			chr3	–	−7.219	4.59E-05	chr3:188665275-188665552	Island	
cg25137968	HIST2H2BA		chr1	+	−7.252	4.43E-05	chr1:120905971-120906396	N_Shore	TSS1500
cg05030450	ODZ2		chr5	+	−7.327	4.08E-05		OpenSea	Body
cg10051200	CPEB4; CPEB4; CPEB4; CPEB4; CPEB4		chr5	–	−7.441	3.61E-05		OpenSea	Body; Body; Body; Body; Body
cg03568094			chr20	+	−7.534	3.27E-05	chr20:61196870-61197072	N_Shelf	
cg14887613	MIER2		chr19	+	−7.674	2.82E-05	chr19:345260-345590	Island	TSS1500
cg18584280			chr15	+	−7.736	2.65E-05		OpenSea	
cg11503661			chr2	+	−7.775	2.54E-05	chr2:242788781-242791121	Island	
cg24239266	OR13C2		chr9	+	−8.256	1.56E-05		OpenSea	1stExon
cg25036067			chr3	+	−9.936	3.36E-06		OpenSea	
cg14469826	ABCC8		chr11	+	−10.509	2.09E-06	chr11:17497463-17498626	S_Shore	TSS1500
cg01097406			chr16	–	−15.204	8.46E-08		OpenSea	
cg27450744			chr8	+	−39.924	1.43E-11	chr8:143645072-143645673	Island	

**Table 2:** Top 10 hypermethylated CpGs with exposure to histological chorioamnionitis (*p* ≤ 0.0001).

**CpG site**	**Gene symbol**	**Gene name**	**Chromosome**	**Strand**	**Test statistics**	**Raw *p*-value**	**Relation to Island**	**Gene location of first annotated transcript**
cg19448065	VGLL4	Vestigial Like Family Member 4	chr3	+	15.189	8.54E-08	OpenSea	Body
cg00995577	AGR2	Anterior Gradient 2, Protein Disulphide Isomerase Family Member	chr7	+	10.897	1.53E-06	OpenSea	TSS1500
cg12363975	AGAP1	ArfGAP With GTPase Domain, Ankyrin Repeat And PH Domain 1	chr2	+	10.709	1.78E-06	OpenSea	Body
cg09304034	SMIM15; CTC-436P18.1	SMIM15 Antisense RNA 1 (aka Small Integral Membrane Protein 15 Antisense RNA 1)	chr5	–	9.534	4.75E-06	N_Shore	5′UTR; TSS1500
cg04381379	CELF4	CUGBP Elav-Like Family Member 4	chr18	+	8.385	1.38E-05	OpenSea	Body
cg02086467	OTX2	Orthodenticle Homeobox 2	chr14	–	7.855	2.34E-05	Island	5′UTR
cg18196614	MCM8-AS1	MCM8 Antisense RNA1	chr20	+	7.710	2.72E-05	N_Shelf	Body
cg04040779	NUP160	Nucleoporin 160	chr11	–	7.439	3.62E-05	OpenSea	Body
cg14508517	NAALADL2	N-Acetylated Alpha-Linked Acidic Dipeptidase Like 2	chr3	–	7.340	4.03E-05	OpenSea	Body
cg15057251	MAPRE2	Microtubule Associated Protein RP/EB Family Member 2	chr18	–	7.306	4.18E-05	Island	TSS200

**Table 3:** Top 10 hypomethylated CpGs with exposure to histological chorioamnionitis (*p* ≤ 0.0001).

**CpG site**	**Gene symbol**	**Gene name**	**Chromosome**	**Strand**	**Test statistics**	**Raw *p*-value**	**Relation to island**	**Gene location of first annotated transcript**
cg14469826	ABCC8	ATP binding cassette subfamily C member 8	chr11	+	−10.509	2.09E-06	S_Shore	TSS1500
cg24239266	OR13C2	Olfactory receptor family 13 subfamily C member 2	chr9	+	−8.256	1.56E-05	OpenSea	1stExon
cg14887613	MIER2	MIER Family Member 2 (aka Mesoderm Induction Early Response 1, Family Member 2)	chr19	+	−7.674	2.82E-05	Island	TSS1500
cg10051200	CPEB4	Cytoplasmic Polyadenylation Element Binding Protein 4	chr5	–	−7.441	3.61E-05	OpenSea	Body
cg05030450	ODZ2	Teneurin Transmembrane Protein 2	chr5	+	−7.327	4.08E-05	OpenSea	Body
cg25137968	HIST2H2BA	Histone Cluster 2 H2B Family Member A (Pseudogene)	chr1	+	−7.252	4.43E-05	N_Shore	TSS1500
cg18901644	GPR78	G Protein-Coupled Receptor 78	chr4	–	−7.030	5.66E-05	Island	TSS200
cg12743398	SULT1A2	Sulfotransferase Family 1A Member 2	chr16	+	−6.953	6.17E-05	N_Shelf	TSS1500
cg26933865	PPP4C	Protein Phosphatase 4 Catalytic Subunit	chr16	+	−6.904	6.52E-05	N_Shore	TSS1500
cg15343100	CCDC68	Coiled-Coil Domain Containing 68	chr18	–	−6.735	7.90E-05	N_Shore	TSS1500

**Figure 2:**
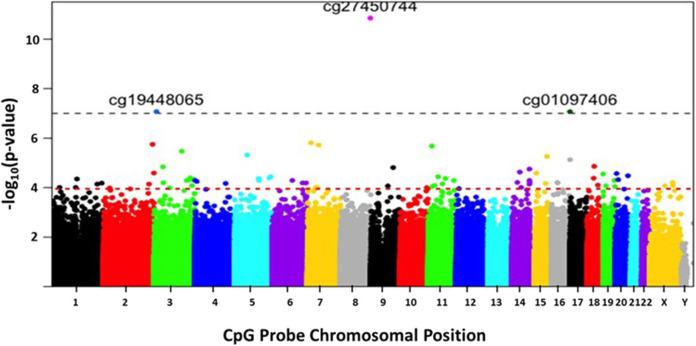
The distribution of differentially methylated CpG sites identified across chromosomes. The x-axis represents the chromosomal location of the CpG probes. The y-axis represents differential methylation as the negative log of the *p*-value of their association. Genome-wide significance threshold of *p* ≤ 0.0001 is used as a cut off (horizontal red line). The black dotted line indicates a significance threshold of *p* ≤ 10^−7^.

**Figure 3:**
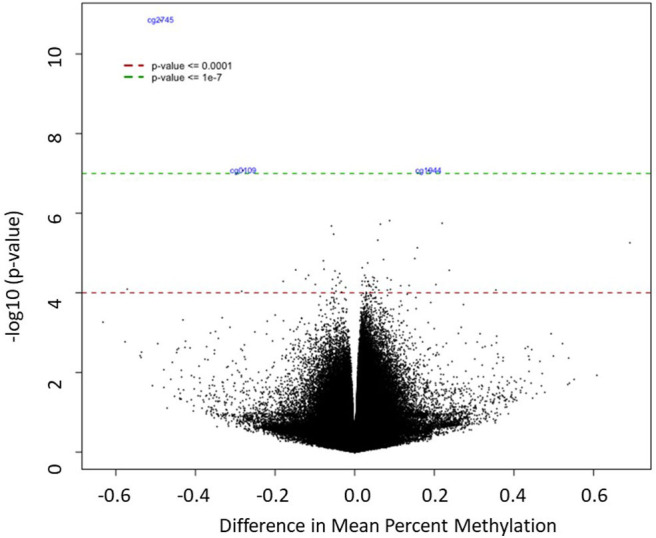
The differentially methylated CpG sites between the HCA and control groups. The x-axis shows difference in mean percent of DNA methylation and the y-axis shows the significance of differential methylation of each probe, represented as the negative log of the *p*-value. The red and green line indicate genome-wide significance thresholds of *p* ≤ 0.0001 and *p* ≤ 10^−7^, respectively.

A Manhattan plot (Figure 2) shows the distribution of possible differentially methylated CpG sites identified across chromosomes. Genome-wide significance threshold of *p* ≤ 0.0001 was used as a cut off (red line). The top candidates in pairwise comparison were selected by test statistics and *p*-values for comparison. The black dotted line indicates a significance threshold of *p* ≤ 10^−7^. Three probe sets including two non-annotated probes (cg27450744, cg01097406) and VGLL4 (cg19448065) remained significant at a threshold of *p* ≤ 10^−7^. The differentially methylated CpG sites are depicted in a volcano plot (Figure 3). A volcano plot is a scatter plot of significance, the negative log of the *p*-value vs. the log of fold change.

### Cluster Analysis – Heatmap

**Figure 4:**
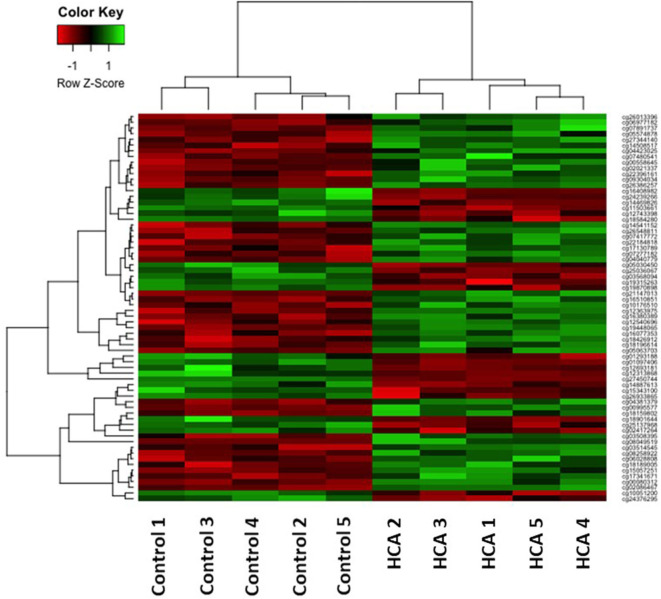
The 68 differentially methylated probe sets (*p* ≤ 0.0001) in the individual HCA samples (right) compared to control samples (left). The green and red colors indicate hyper-methylated and hypo-methylated loci, respectively.

Cluster analysis was performed on the 68 differentially methylated loci for the two sample groups using the hierarchical clustering method. A heatmap of the methylation levels for the 68 DNA methylation loci illustrates the differences between the two groups (Figure 4). The hypermethylated loci are depicted in green, and the hypomethylated loci are depicted in red. Each column in the heatmap represents a sample.

### Differential DNA Methylation Related to Immune Response

Eleven genes related to immune response and regulation of inflammatory pathways were identified (Table 4). Seven genes were hypermethylated and 4 genes were hypomethylated. The hypermethylated genes have roles in asthma (VGLL4, AGR2) (24, 28), neurologic development (AGAP1, OTX2) (29, 30), and immune regulation (SCAMP5, SLC11A1) (31, 32). The hypomethylated genes have roles in immune regulation (PPP4C) (27), transcription regulation (MIER2) (26), and inflammation (ABCC8) (33).

**Table 4:** Key genes related to immune response with exposure to histological chorioamnionitis (*p* ≤ 0.0001).

**CpG site**	**Gene symbol**	**Chromosome**	**Strand**	**Test statistics**	**Methylation status**	**Raw *p*-values**	**Relation to Island**	**Gene location/Reference gene group**
cg19448065	VGLL4; VGLL4	chr3	+	15.189	Hyper-methylated genes	8.54E-08	OpenSea	Body; Body
cg00995577	AGR2	chr7	+	10.897		1.53E-06	OpenSea	TSS1500
cg12363975	AGAP1; AGAP1; AGAP1	chr2	+	10.709		1.78E-06	OpenSea	Body; Body; Body
cg02086467	OTX2	chr14	–	7.855		2.34E-05	Island	5′UTR
cg14541152	SCAMP5	chr15	–	6.876		6.73E-05	N_Shelf	3′UTR
cg17130789	SLC11A1	chr2	–	6.806		7.29E-05	Island	Body
cg26933865	PPP4C	chr16	+	−6.904	Hypo-methylated genes	6.52E-05	N_Shore	TSS1500
cg14887613	MIER2	chr19	+	−7.674		2.82E-05	Island	TSS1500
cg14469826	ABCC8	chr11	+	−10.509		2.09E-06	S_Shore	TSS1500

### Ingenuity Pathway Analysis

Ingenuity Pathway Analysis (IPA) software (Qiagen Inc., Germantown, MD) was used to perform pathway analysis by loading 448 probe sets that were found to be differentially methylated with exposure to HCA for a *p* ≤ 0.001. A total of 76 diseases and functions were altered with exposure to HCA. Selected key diseases and functions altered with HCA are listed in Table 5. The modification of functions with HCA included genes related to nervous system development and function, inflammatory response and disease, cellular movement, death and survival, hematological system development and function, and respiratory disease, development and function. IPA identified 224 canonical pathways that were modified with exposure to HCA. Selected key pathways important in immune regulation and inflammatory responses are shown in Table 6.

**Table 5:** Diseases and functions modified with exposure to histological chorioamnionitis.

**Modified diseases and functions**	***p*-value**	**Number of molecules involved**	**Molecules involved**
Nervous system development and function	3.08E-04 to 2.03E-02	30	LINGO2,GRM3,MYT1L,SPTBN1,PTN,NLGN1,IL1R1,PIK3CA,HMGB1,PDE11A,CNTNAP2,HS6ST1,ELMO1, HSF1,LRRC4C,SCAMP5,SHANK1,ALK,OTX2,TENM2,RMST,IRX6,RAB21,NMU,ATP7A,KLF7,CCND1,PRKAA2,ARHGAP32,LRRK2
Inflammatory response	3.09E-04 to 1.58E-02	10	HMGB1,IL1R1,ATG5,PDE11A,CCND1,TUBB1,ALK,SOS1,TRNT1,HSF1
Inflammatory disease	3.82E-04 to 1.58E-02	8	IL1R1,HMGB1,NAALADL2,PDE11A,COL28A1,CSMD1,CACNA2D3,TUBB1
Cell death and survival	1.01E-03 to 2.03E-02	17	SF3B1,TBP,ATG5,TUB,ABCC1,OTX2,CD70,GALNT3,HMGB1,IL1R1,CCND1,PRKAA2,MEIS1,MYSM1,LRRK2,ELMO1,HSF1
Cellular movement	1.01E-03 to 1.91E-02	16	LDLRAP1,RECK,ALK,SOS1,IGF2R,SYT7,HAS2,HMGB1,PIK3CA,IL1R1,CCND1,PRKAA2,IL19,RAMP3,ELMO1,ETV6
Hematological system development and function	1.01E-03 to 2.03E-02	8	NMU,HMGB1,PIK3CA,CCND1,TUBB1,TMOD3,TSPAN33,SOS1
Immune cell trafficking	1.01E-03 to 2.03E-02	4	HMGB1,PIK3CA,CCND1,SOS1
Neurological disease	1.05E-03 to 2.03E-02	32	TBP,SF3B1,TUBB1,CSNK1G2,GRM3,MYT1L,RPL5,KANSL1,PIK3CA,IL1R1,KIAA0556,CNTNAP2,HS6ST1,SACS,ELMO1, HSF1,BRAT1,RECK,TAF2,ALK,ACO2,SLC25A16,NALCN,ABCC1,OTX2,TENM2,NAV3,ATP7A,KLF7,CCND1,FAM174B,LRRK2
Cellular development	1.34E-03 to 1.74E-02	43	ATG5,FGF19,DOCK4,ELF2,LINGO2,GRM3,MYT1L,SPTBN1,PTN,HAS2,NLGN1,IL1R1,PIK3CA,HMGB1,BATF2,CNTNAP2, HS6ST1,TSPAN33,ELMO1,HSF1,LRRC4C,LDLRAP1,RECK,TNK1,SHANK1,ALK,MAGED1,OTX2,SOS1,CD70,GALNT3,RMST,I RX6,RAB21,XPO1,ATP7A,KLF7,CCND1,PRKAA2,ARHGAP32,LRRK2,ETV6,LTBP1
Cellular growth and proliferation	1.34E-03 to 2.03E-02	51	ATG5,FGF19,ELF2,GRM3,IFT52,HMGB1,CNTNAP2,HS6ST1,TSPAN33,HSF1,LRRC4C,LDLRAP1,RECK,ALK,CD70,GALNT3, IRX6,RAB21,XPO1,ATP7A,CCND1,RASGRF2,MEIS1,LRRK2,DOCK4,LINGO2,AGR2,MYT1L,SPTBN1,PTN,HAS2,NLGN1,IL1R1, PIK3CA,BATF2,HDAC4,ELMO1,TNK1,SHANK1,AICDA,MAGED1,ABCC1,OTX2,SOS1,RMST,KLF7,AQP2,PRKAA2,ARHGAP32,ETV6,LTBP1
Respiratory disease	2.1E-03 to 2.03E-02	18	SF3B1,CSMD1,TUBB1,AICDA,ALK,OTX2,GRM3,NAV3,MYF5,RPL5,HMGB1,IL1R1,PIK3CA,CCND1,CNTNAP2,LRRK2,MEGF11,HSF1
Immunological disease	2.17E-03 to 2E-02	40	SF3B1,TBP,ATG5,TUBB1,ELF2,CSNK1G2,ZMIZ1,GRM3,SPTBN1,KLHL24,RPL5,HMGB1, PIK3CA,PDE11A,IL1R1,CNTNAP2,HDAC4,ELMO1,TRNT1,LRRC1,FAM189A1,TNK1,SHANK1, AICDA,ALK,NALCN,ABCC1,TENM2,CD70,OTX2,SOS1,NAV3,EYS,XPO1,CCND1,EIF2A, TPST2,LRRK2,ETV6,LTBP1
DNA replication, recombination, and repair	2.18E-03 to 1.58E-02	8	SNX13,XPO1,TBP,PIK3CA,HMGB1,PDE11A,PRKAA2,ABCC1
Cellular function and maintenance	2.78E-03 to 1.75E-02	43	DYM,VPS37D,ATG5,TUB,DOCK4,GMNC,BHLHA15,SPTBN1,KLHL24,IFT52,HAS2,PTN,NLGN1,PIK3CA,HMGB1,IL1R1,WDR41, TESK2,CNTNAP2,TMOD3,NUDCD3,HDAC4,ELMO1,HSF1,LRRC4C,BRAT1,ABCC1,NUP160,UBASH3B,NAV3,CLIP3,RAB21,XPO1, ATP7A,KLF7,CCND1,EIF2A,RAB32,PRKAA2,ARHGAP32,KIF18B,LRRK2,KDM4A
Respiratory system development and function	2.78E-03 to 2.03E-02	4	MYF5,HMGB1,OTX2,HSF1
Cell cycle	2.82E-03 to 2.03E-02	27	ATG5,CDK14,TUBB1,MYF5,HAS2,PTN,HMGB1,PIK3CA,SACM1L,TMOD3,NUDCD3,HDAC4,HSF1,RECK,CENPH,ALK,ANKS1A, MAGED1,CD70,XPO1,CCND1,MEIS1,ARHGAP32,MCIDAS,KIF18B,LRRK2,LTBP1
Cell-to-cell signaling and interaction	3.76E-03 to 2.03E-02	17	ATG5,FGF19,PTS,ALK,MAGED1,IGF2R,GRM3,RAB21,HAS2,NMU,NLGN1,HMGB1,IL1R1,PDE11A,PIK3CA,CNTNAP2,LRRK2
Cell-mediated immune response	1.02E-02 to 1.02E-02	1	PIK3CA
Gene expression	1.02E-02 to 2.03E-02	2	TBP,HMGB1

**Table 6:** Canonical pathways in Ingenuity Pathway Analysis associated with exposure to histological chorioamnionitis.

**Ingenuity canonical pathways**	**–log(*p*-value)**	**Molecules involved in pathways**	**Importance**
CREB signaling in neurons	2.23	TBP,PIK3CA,GRID1,CACNA2D3,CACNG3,SOS1,GRM3	Neuronal protection
FcγRIIB signaling in B lymphocytes	2.06	PIK3CA,CACNA2D3,CACNG3,SOS1	Apoptosis, autoimmunity
Cell cycle: G1/S checkpoint regulation	1.54	RPL5,CCND1,HDAC4	Cell cycle regulation
HGF signaling	1.53	PIK3CA,CCND1,ELF2,SOS1	T-cell related
Tetrahydrobiopterin biosynthesis I	1.52	PTS	Alters neurotransmitters
Tetrahydrobiopterin biosynthesis II	1.52	PTS	Alters neurotransmitters
GM-CSF signaling	1.41	PIK3CA,CCND1,SOS1	Immune response
3-phosphoinositide biosynthesis	1.31	SGPP1,PIK3CA,UBLCP1,PPP4C,SACM1L	Cell cycle regulation
Glucocorticoid receptor signaling	1.24	GTF2H3,TBP,HMGB1,PIK3CA,TAF2,PRKAA2,SOS1	Inflammation/Stress
IL-7 signaling pathway	1.24	PIK3CA,CCND1,SOS1	Immune response
FGF signaling	1.18	PIK3CA,FGF19,SOS1	Cell proliferation
3-phosphoinositide degradation	1.15	SGPP1,UBLCP1,PPP4C,SACM1L	Cell cycle regulation
Role of NFAT in cardiac hypertrophy	1.15	PIK3CA,CACNA2D3,CACNG3,HDAC4,SOS1	Immune modulation
PKCθ signaling in T lymphocytes	1.14	PIK3CA,CACNA2D3,CACNG3,SOS1	Immune response
G beta gamma signaling	0.955	CACNA2D3,CACNG3,SOS1	Inflammation
Melatonin degradation I	0.953	SULT1A2,CYP2S1	CNS
Glutamate receptor signaling	0.953	GRID1,GRM3	CNS
Role of NFAT in regulation of the immune response	0.943	XPO1,PIK3CA,CSNK1G2,SOS1	Immune response
CNTF signaling	0.904	PIK3CA,SOS1	CNS
Superpathway of melatonin degradation	0.892	SULT1A2,CYP2S1	CNS
PI3K/AKT signaling	0.883	PIK3CA,CCND1,SOS1	Cell cycle regulation
IL-2 signaling	0.858	PIK3CA,SOS1	Immune response
IL-6 Signaling	0.846	PIK3CA,IL1R1,SOS1	Immune response
HMGB1 signaling	0.825	HMGB1,PIK3CA,IL1R1	Inflammation
LPS/IL-1 mediated inhibition of RXR function	0.782	XPO1,IL1R1,SULT1A2,HS6ST1	Inflammation
GADD45 signaling	0.752	CCND1	Cell cycle regulation
GDNF family ligand-receptor interactions	0.741	PIK3CA,SOS1	CNS
Neurotrophin/TRK signaling	0.732	PIK3CA,SOS1	CNS
IL-17A signaling in airway cells	0.724	PIK3CA,IL19	Inflammation
Cyclins and cell cycle regulation	0.715	CCND1,HDAC4	Cell cycle regulation
IL-3 signaling	0.683	PIK3CA,SOS1	Immune response
FLT3 signaling in hematopoietic progenitor cells	0.683	PIK3CA,SOS1	Immune response
IL-17 signaling	0.668	PIK3CA,IL19	Inflammation
PPAR signaling	0.618	IL1R1,SOS1	Transcription factor
PPARα/RXRα activation	0.61	IL1R1,PRKAA2,SOS1	Transcription factor
Dopamine degradation	0.577	SULT1A2	CNS
T cell receptor signaling	0.521	PIK3CA,SOS1	Immune response
p53 signaling	0.505	PIK3CA,CCND1	Cell proliferation
Serotonin receptor signaling	0.456	PTS	CNS
Serotonin degradation	0.317	SULT1A2	CNS
IL-10 signaling	0.295	IL1R1	Inflammation
Wnt/β-catenin signaling	0.288	CCND1,CSNK1G2	Cell Signaling
Dopamine receptor signaling	0.266	PTS	CNS
Regulation of IL-2 expression in activated and anergic T lymphocytes	0.255	SOS1	Immune response
B cell receptor signaling	0.242	PIK3CA,SOS1	Immune response
IL-8 signaling	0.225	PIK3CA,CCND1	Inflammation
IL-1 signaling	0.214	IL1R1	Inflammation
CTLA4 Signaling in Cytotoxic T Lymphocytes	0.198	PIK3CA	Immune response

IPA identified 15 networks, of which network 2 (cellular growth and proliferation, cellular development, connective tissue development and function) and network 13 (cellular movement, hematological system development and function, immune cell trafficking) are closely related to the focus of this study.

## Discussion

Exposure to HCA is associated with an increased risk of medical problems later in life including cerebral palsy, developmental delay, asthma, and allergic disorders (6–13). Although the mechanism of this increased risk is not yet known, alteration in gene expression secondary to differential DNA methylation may contribute to this increased risk. To our knowledge, this is the first study to report differential DNA methylation in human cord blood mononuclear leukocytes in term neonates exposed to HCA. Our data suggest that HCA altered the methylation status of genes involved in inflammation, immune regulation, respiratory development, and neurologic development in cord blood mononuclear leukocytes from term neonates.

Infants born to mothers with HCA are at increased risk for asthma but the exact mechanism is unknown (6, 7, 11, 12). Our results show that exposure to HCA was associated with differential methylation of several genes involved in signaling pathways related to the lung development, lung inflammation, and asthma. Vestigial Like Family Member 4 (VGLL4) is a gene related to Wnt/β-catenin signaling pathway regulation, cell cycle regulation, and immune regulation via T-cell mediated tumor regression (28, 34, 35). The Wnt/β-catenin signaling pathway plays a pivotal role in lung development, lung injury, and repair (28). Aberrant expression of Wnt/β-catenin signaling can lead to asthmatic airway remodeling with hyperplasia of airway smooth muscle cells, goblet cell metaplasia, and extracellular matrix deposition (28). Down-regulation of VGLL4 due to hypermethylation after exposure to HCA could lead to dysregulation of Wnt/β-catenin signaling, abnormal lung development, and an increased risk for asthma.

Cyclin D-1 gene (CCND1), another regulator of the Wnt/β-catenin signaling pathway, is differentially methylated after exposure to HCA and associated with airway remodeling and smooth muscle proliferation in asthma (36, 37). Mutation in the ATP Binding Cassette Subfamily C Member 8 (ABCC8) gene is associated with pulmonary arterial hypertension (38). Receptor Activity-Modifying Protein 3 (RAMP3), the top hypomethylated gene with exposure to HCA, plays a role in human lung development (39). An additional top hypermethylated gene is Anterior Gradient 2 (AGR2), a protein disulfide isomerase family member known to be involved in asthma and allergen-induced mucin production (24).

Previous studies have also shown that HCA is associated with neurodevelopmental impairment and cerebral palsy (8–10, 13). Several genes involved in nervous system development and function, neurological diseases, and neurological pathways were found to be differentially methylated in cord blood mononuclear leukocytes after exposure to HCA. Hypermethylated genes involved in neurological disorders include AGAP1, CACNA1A, and OTX2. AGAP1 is known to be involved in dopamine regulation and has been shown to increase endocytic recycling of M5 muscarinic receptors involved in dopamine release in the midbrain (29). Recent work by Pacault et al. showed that deletion of AGAP1 is reported in a patient with autism (40). Genetic variation in the CACNA1A gene is associated with cerebral palsy (41). OTX2 mutations are associated with severe ocular phenotypes, seizures, and developmental delay (30).

Examples of hypomethylated genes in our study include ABCC8, MIER2, and PPP4C. ABCC8 has been shown to be associated with inflammation regulation in the nervous system in autoimmune encephalomyelitis (33). MIER2 is involved in recruitment of histone deacetylase complexes (26). PPP4C plays a key role in cortical neurodevelopment by ensuring symmetric division of early cortical cells (42). Absence of PPP4C has been shown to result in early asymmetric division and premature neurogenesis. Hypomethylation of PPP4C may result in abnormal cortical development, increasing the risk of neurodevelopmental disorders. PPP4C has also been shown to be involved in immune regulation through proliferation and expansion of regulatory T cells and subsequent adaptive immunity (27). We speculate that the fetal systemic inflammatory response from HCA leads to changes in methylation pattern in leukocytes. The systemic inflammatory response outside the fetal brain can induce neuroinflammation and neuronal injury (43, 44). The inflammatory response in fetal brain due to HCA may change the DNA methylation pattern in nervous system cells.

We report differential DNA methylation in genes involved in lung development, neurodevelopment, immune regulation, and inflammation after exposure to HCA. Changes in DNA methylation may result in differential gene expression and alter these processes throughout life, being a potential mechanism for the increased risk of disorders seen with exposure to HCA. Our hierarchical clustering method analysis showed extremely consistent results across the samples from each group suggesting high reliability of the differential methylation patterns that we found. Furthermore, IPA identified diseases and functions that were consistent with the known associations of HCA, including modification of nervous system development and function, inflammatory response and disease, as well as respiratory disease, development, and function.

Our study has multiple strengths. This is the first study reporting differential DNA methylation of cord blood mononuclear leukocytes in term neonates born to mothers with HCA. Genome-wide DNA methylation was performed on the mononuclear leukocytes rather than whole blood. We chose umbilical cord blood which allowed us to obtain a significant volume of blood with no invasive risk to the neonate. We also recognize several limitations in our study. The sample size of 10 neonates is small, although similar sample sizes have been used in prior studies of differential DNA methylation (45). There is a chance of finding differential gene expression when comparing such a high number of genes but that was mitigated by using a very stringent *p*-value. Another weakness of the study is a lack a validation cohort. A larger study to confirm our finding is needed.

## Conclusions

Exposure to HCA results in differential DNA methylation of cord blood mononuclear leukocytes in term neonates. This differential DNA methylation was present in genes involved in immune modulation, inflammation, lung development, and nervous system development. Future studies are needed to further validate these results in a larger group of neonates and by examining functional differences related to these genes. Our data may lead to an improved understanding of the genes and pathways involved in exposure to HCA. Identifying and understanding the role of novel candidate genes can lead to the development of prognostic biomarkers and therapies to mitigate the long-term consequences of abnormal DNA methylation induced by HCA.

## Data Availability Statement

The raw data are available at the Gene Expression Omnibus (GEO) database of the NIH, under accession number GSE153668.

## Ethics Statement

The studies involving human participants were reviewed and approved by Institutional Review Board of Thomas Jefferson University Hospital and the Nemours Institutional Biosafety Committee. Written informed consent for participation was not required for this study in accordance with the national legislation and the institutional requirements.

## Author's Note

Presented in part at the Eastern Society for Pediatric Research Annual Meeting; Philadelphia, PA; March 2018 and the Annual Meeting of the Pediatric Academic Societies; Toronto, Canada; May 2018.

## Author Contributions

GF, SG, and SM contributed equally to concept and design, sample collection and processing, acquisition and assembly of data, data analysis and interpretation, and manuscript writing. MF contributed to sample collection, acquisition and analysis of data, and manuscript writing. JC and SA contributed to data analysis and interpretation. VB, TS, and JG contributed to concept and design and data analysis and interpretation. IR contributed to concept and design, interpretation, and manuscript editing. ZA contributed to concept and design, data analysis and interpretation, and manuscript writing and editing. All authors have approved the version of the submitted manuscript.

## Conflict of Interest

The authors declare that the research was conducted in the absence of any commercial or financial relationships that could be construed as a potential conflict of interest.
