# Supplementary material for: DNA Methylation Profile in Human Cord Blood Mononuclear Leukocytes From Term Neonates: Effects of Histological Chorioamnionitis
Source: Front Pediatr. 2020 Aug 4;8:437. doi: 10.3389/fped.2020.00437 (PMC7417608; doi:10.3389/fped.2020.00437)
Supplement: Supplementary file 1 [file Data_Sheet_1.PDF]

Supplemental Figure 1: The stage and grade of HCA (Redline et al., 2003)

**Sample number**	**Stage**	**Grade**
#1	1	1
#2	1	1
#3	2	2
#4	2	1
#5	2	1

Stage: 1 = acute subchorionitis or chorionitis, 2 = acute chorioamnionitis, 3 = necrotising chorioamnionitis

Grade = 1: not severe as defined by Grade 2; 2: confluent PMN between chorion and decidua; 3: isolated foci or continuous band
